# Pheromone Diversification and Age-Dependent Behavioural Plasticity Decrease Interspecific Mating Costs in *Nasonia*

**DOI:** 10.1371/journal.pone.0089214

**Published:** 2014-02-14

**Authors:** Joachim Ruther, Jennifer McCaw, Lisa Böcher, Daniela Pothmann, Irina Putz

**Affiliations:** Institute for Zoology, University of Regensburg, Regensburg, Germany; University of Arkansas, United States of America

## Abstract

Interspecific mating can cause severe fitness costs due to the fact that hybrids are often non-viable or less fit. Thus, theory predicts the selection of traits that lessen reproductive interactions between closely related sympatric species. Males of the parasitic wasp *Nasonia vitripennis* differ from all other *Nasonia* species by an additional sex pheromone component, but the ecological selective forces underlying this pheromone diversification are unknown. Here we present data from lab experiments suggesting that costly interspecific sexual interactions with the sympatric species *N. giraulti* might have been responsible for the pheromone evolution and some courtship-related behavioural adaptations in *N. vitripennis.* Most *N. giraulti* females are inseminated already within the host, but *N. giraulti* males still invest in costly sex pheromones after emergence. Furthermore, they do not discriminate between *N. vitripennis* females and conspecifics during courtship. Therefore, *N. vitripennis* females, most of which emerge as virgins, face the risk of mating with *N. giraulti* resulting in costly all-male broods due to *Wolbachia*-induced cytoplasmic incompatibility. As a counter adaptation, young *N. vitripennis* females discriminate against *N. giraulti* males using the more complex conspecific sex pheromone and reject most of them during courtship. With increasing age, however, *N. vitripennis* females become less choosy, but often compensate mating errors by re-mating with a conspecific. By doing so, they can principally avoid suboptimal offspring sex ratios, but a microcosm experiment suggests that under more natural conditions *N. vitripennis* females cannot completely avoid fitness costs due to heterospecific mating. Our study provides support for the hypothesis that communication interference of closely related sympatric species using similar sexual signals can generate selective pressures that lead to their divergence.

## Introduction

Sexual interactions with heterospecifics are often costly for sexually reproducing organisms. Mating with a sexual partner from another species, for instance, may result in dramatic fitness losses because hybrids may be non-viable or less fit than offspring resulting from intraspecific fertilization [1]. Therefore, theory predicts the selection of traits that lessen costly reproductive interactions with sympatric heterospecifics thus favouring assortative mating between conspecifics. Depending on the communicative channels involved in mate finding and pre-copulatory sexual signalling, this process is typically accompanied by a divergence of visual, acoustic, tactile and olfactory traits, which mediate premating isolation and may thus contribute to speciation [2], [3].

In insects, the chemical sense is of particular importance for sexual communication and many of them use volatile sex pheromones to attract potential mates from a distance whereas less volatile compounds are used at close range for mate recognition and to elicit receptivity during courtship [2], [4]. Many congeneric species share the same sex pheromone components [5], which in sympatry may lead to costly interference of sexual communication. Thus, interspecific divergence of chemical traits has been reported for a number of sympatric species (reviewed by [2]), but the causal link between diverged chemical traits and the ability of species to discriminate against heterospecifics is often missing. Furthermore, the genetic mechanisms as well as the ecological selective forces underlying signal diversification have hitherto been studied only in relatively few species mostly from the orders Lepidoptera and Diptera [2].

In the present study, we investigated interspecific sexual interactions in the parasitic wasp genus *Nasonia.* The genus comprises four species, i.e. *N. vitripennis* (*Nv*), *N. giraulti* (*Ng*), *N. longicornis* and *N. oneida*, which parasitise pupae of numerous fly species [6], [7]. *Nv* is a cosmopolitan species whereas the occurrence of *Ng* and *N. longicornis* is restricted to the eastern (*Ng*) and western (*N. longicornis*) part of North America [8]. *N. oneida* has hitherto only been reported from the state of New York [6] where it co-occurs with *N. vitripennis* and *N. giraulti* [6], [9]. The typical natural habitat of *Nasonia* wasps is nests of hole-breeding birds [8]. *Nasonia* wasps are haplodiploid species with female offspring developing from fertilised eggs and male offspring from unfertilised ones. In nature, *Nasonia* females typically mate only once in their lifetime [10] and subsequently disperse to search for new host patches. The mating system of *Nasonia* species is characterised by local mate competition [11] with a high degree of sib mating at the natal patch. Therefore, females exploiting a given host patch alone produce only the minimum number of sons necessary to fertilise all their sisters. With increasing number of foundresses, offspring sex ratios are shifted in favour of sons because of increasing competition between non-sib males [12]. In natural habitats, however, typically strongly female-biased sex ratios are found [13], [14]. Hence, any factors constraining females to produce more males than necessary or even all-male broods imply fitness costs and should be selected against. One such factor in *Nasonia* is interspecific mating, which is possible in principle, but hybrids are not formed in most combinations due to *Wolbachia*-mediated cytoplasmic incompatibility. Females mating with a heterospecific male produce all-male broods because of improper condensation of the paternal chromosomes [15]. Hence, the evolution of mechanisms that reduce the risk of interspecific mating between sympatric *Nasonia* species is expected.

Males of all *Nasonia* species produce sex pheromones in their rectal vesicle to attract virgin females [16]–[18]. In *Nv,* the response of females to the male sex pheromone depends on their mating status. Mated females no longer respond to the male sex pheromone [16], [19], but it is unknown whether this behavioural switch occurs also after heterospecific mating. The composition of the male sex pheromone varies between the *Nasonia* species [18]. All species produce the two sex pheromone components (4*R*,5*S*)-5-hydroxy-4-decanolide (*RS*) and 4-methylquinazoline (MQ), but only *Nv* has evolved a third component, (4*R*,5*R*)-5-hydroxy4-decanolide (*RR*). *Nv* females use *RR* to discriminate between the three-component pheromone of *Nv* males and the less complex blend of the other species [18]. Three genes coding for short chain dehydrogenases/reductases (SDRs) [20] are responsible for the pheromone difference between *Nv* and the other *Nasonia* species. The SDRs are assumed to invert the stereochemistry at carbon atom five of 5-hydroxy4-decanolide (HDL) and knock-down of the genes by RNAi results in the loss of *RR* in the *Nv* pheromone blend [18]. Hence, the genetic and biochemical basis for the pheromone diversification in *Nv* have been widely clarified, but the selective ecological forces which have driven the evolution of *RR* are only poorly understood.

Like most parasitoids, *Nasonia* species perform stereotypic courtship behaviour prior to mating, in which at least two further pheromones are involved. When perceiving the female cuticular hydrocarbons (CHCs), males are arrested, subsequently mount the female and show so-called head nodding behaviour [21], which serves the release of a still unknown aphrodisiac pheromone from an oral gland. The release of this pheromone is a prerequisite for females to become receptive [22], [23]. Semiochemical-based mate recognition during courtship appears to be one mechanism of premating isolation in *Nasonia* because mating rates of interspecific couples are typically much lower than those of intraspecific ones [24], [25]. However, given that interspecific mating occurs, neither the female CHCs nor the male aphrodisiac appear to convey information in a strictly species specific manner. In accordance with this assumption, a varying degree of male cross responsiveness to female cuticular extracts has been found in interspecific mate recognition trials [9].

In the present study, we tested the hypothesis that costs associated with interspecific mating among sympatric *Nasonia* species might have caused the chemical diversification in the *Nv* sex pheromone blend. In our experiments, we focused on reproductive interactions between *Nv* and *Ng* because these two species are regularly found micro-sympatrically in eastern North America, i.e., they regularly develop within the same hosts [8], [13], [24]. Furthermore, the two species differ in an important detail of their reproductive behaviour in that *Ng* shows a high degree of within-host mating whereas the majority of *Nv* females are inseminated outside the host [26]. Considering that male and female *Ng* eclose very close to each other within the host and also mate at this site, we predict that the male sex pheromone is widely dispensable for mate finding in *Ng.* We demonstrate that *Ng* males invest nonetheless in pheromone production and hypothesise that this has led to communication interference with *Nv* in ancestral times resulting in pheromone diversification in *Nv* males and a species-specific pheromone response in females. We predict furthermore the evolution of courtship-related behavioural adaptations in *Nv* females to avoid interspecific mating with *Ng* males both within multiparasitised hosts and shortly after emergence outside the host. We tested these predictions in a comprehensive series of lab experiments combining chemical analyses, pheromone bioassays and mating trials.

## Materials and Methods

### Insects

*Nv* originated from the inbred strain Phero01; *Ng* originated from the inbred strain NGVA2 and were kindly provided by Thomas Schmitt (University of Würzburg, Germany). Both species were reared on freeze-killed puparia of the green bottle fly *Lucilia caesar* [21]. To obtain wasps of defined age and mating status for the experiments, parasitoid pupae were excised from host puparia 1–2 days prior to eclosion and kept singly in 1.5 ml microcentrifuge tubes until emergence.

### Degree of within Host Mating in *Ng* and *Nv*

In this experiment we tested the degree of within host mating in the two *Nv* and *Ng* strains used for this study. Owing to the haplodiploidy in *Nasonia*, the presence of daughters in the offspring of a given female indicates her successful insemination. Females of either species (n = 20 per species) were collected in the moment of emergence from the host. Mating outside the host was carefully prevented. Hence, insemination of the females, if any, must have taken place inside the host. Subsequently, females were individually exposed to two hosts for 48 h and the emerging offspring were checked for the presence of daughters. Ratios of within host mating were compared by 2×2 Χ^2^-test.

### Pheromone Quantification

To investigate whether *Nv* and *Ng* differ in their investment in pheromone production, we analysed HDL titres of 2-d-old males (n = 10 for each species). Male wasps were frozen (−20°C) and dissected abdomens were extracted for 30 min with 25 µl dichloromethane containing 10 ng µl^−1^ methyl undecanoate as an internal standard. Quantification of HDL was done by gas chromatography coupled to mass spectrometry (GC-MS) using the protocol and instrumentation described previously [27]. To determine the earliest possible point in time when *Ng* males may use their abdominal sex attractant, we investigated the age dependency of pheromone production in *Ng* males by analysing newly emerged (referred to as 0-d), 1-d, 2-d and 3-d-old males (n* = *5 per group) using the same method. Finally, we investigated whether *Ng* males release HDL inside the host during the period of within host mating. For this purpose, we analysed *Ng-*parasitised host puparia from which all wasps had emerged (n = 20) with the method described above, but used 50 µl of the internal standard solution for extraction. Total HDL amounts in *Ng* and *Nv* males were compared by a *t*-test for independent data. Age dependency of HDL titres was analysed by a Kruskal-Wallis *H-*test followed by Holm-corrected multiple Mann-Whitney *U-*tests for individual comparisons.

### Preparation of Pheromone Extracts for the Bioassays

Sex pheromone extracts of either species were obtained by extracting batches of 40–100 abdomens of 2-d-old frozen males with 20 µl per abdomen for 30 min. Raw extracts were concentrated to 500 µl and cleaned-up by adsorption chromatography as described elsewhere [16]. Fractions were adjusted to a final concentration of 50 ng µl^−1^ and kept at −20°C before being used in the bioassays.

### Pheromone Bioassays

#### General design of the bioassay

The responses of *Ng* and *Nv* females to con- or heterospecific sex pheromone extracts were examined using a dual-choice olfactometer [18] (Figure S1). It consisted of a round glass arena (9 cm diameter) with an hole (3 mm diameter) in the centre to release the wasps, four symmetrically arranged spherical cavities (1 cm diameter, 4 mm depth, 3 cm distance from the central hole) for sample storage and a plastic rim (2 mm height) as distance holder. Two µl of pheromone extract (representing 100 ng of total HDL) or the pure solvent (control) was applied to discs of filter paper (5 mm diameter). After an evaporation time of one minute, paper discs were put into two opposing cavities of the olfactometer (the remaining two cavities remained unused in these tests), a female wasp was released into the central hole and the arena was covered with a glass plate. To avoid the influence of optical stimuli, an open grey plastic cylinder (11 cm diameter, 6 cm height) was placed around the arena and the set-up was illuminated from above with a lamp. Residence time spent in test and control cavity was recorded for 5 min using The Observer XT 9.0 observational software (Noldus, Wageningen, The Netherlands) (n* = *20 for each test). Residence times of females spent in test and control cavities were analysed by a Wilcoxon matched-pairs test. Wasps were tested only once. The olfactometer was thoroughly cleaned with ethanol after every replicate.

#### Bioassay 1

This test aimed at investigating whether *Ng* females respond to the sex pheromone of conspecific males. The response of three types of *Ng* females to the *Ng* pheromone extract was tested: (a) virgin, (b) mated under controlled conditions with a conspecific male or (c) collected in the moment of emergence from the host.

#### Bioassay 2

This test was done to investigate whether *Nv* females are attracted to the sex pheromone of *Ng* males. Thus, the test was carried out exactly like Bioassay 1 but using *Nv* females.

#### Bioassay 3

The results of Bioassay 2 (see below) suggested that the attraction of *Nv* females to the *Ng* pheromone is age-dependent. To verify this under controlled conditions, the response of virgin *Nv* females of different ages (0-d, 1-d and 2-d) to an *Ng* pheromone extract was tested as described above.

#### Bioassay 4

This test aimed at investigating whether mating with *Ng* males and the occurrence of *Ng* males within the same host influence the attraction of *Nv* females to their own pheromone. The response of three types of *Nv* females to an *Nv* pheromone extract was tested: (a) virgin; (b) mated under controlled conditions with an *Ng* male; (c) collected in the moment of emergence from the host that contained only conspecifics; (d) collected in the moment of emergence from the host that contained conspecifics and additionally *Ng* males. For experiment (b), pairs of 2-d-old virgin *Nv* females and *Ng* males were observed in an observation chamber until mating occurred. Couples that did not mate within the first 5 minutes were discarded. To obtain hosts in which *Nv* females and *Ng* males develop at the same time for experiment (c), we exposed pairs of two hosts simultaneously to one mated *Nv* and one virgin *Ng* female each. After 24 h, the females were removed and hosts were kept at rearing conditions until parasitoid emergence. *Nv* females were collected in the moment of emergence from the host as described above.

### Mate Recognition Bioassay

This experiment aimed at elucidating whether *Ng* males are likely to involve *Nv* females in interspecific courtship in zones of sympatry. Courtship behaviour in *Nasonia* can be initiated by exposing males to freshly killed females (“dummies”) [9], [21]. To test whether courting *Ng* males discriminate between conspecific and *Nv* females, we tested their response to 1-d-old *Ng* and *Nv* females, which had been killed by freezing and thawed for 30 min at room temperature. Dummies were fixed in an upright position on a piece of white paper using a droplet of non-toxic superglue (Pattex, Henkel AG, Düsseldorf, Germany). Responses of *Ng* males were tested toward single dummies (no choice) or pairs of two (choice between *Ng* and *Nv*) in a round observation arena (10 mm diameter×3 mm height). The residence time of males on the dummies and copulation attempts were recorded for an observation time of 5 min. When pairs were tested, it was additionally noted which of the two dummies was mounted first (first choice). Residence time was analysed by a *t*-test for independent (no-choice test) and dependent samples (choice test), respectively; first choice was analysed by a Χ^2^-test for the goodness of fit (assuming equal distribution as null hypothesis) and the proportion of males showing copulation attempts by a two-sided Fisher’s exact test (n* = *20).

### Mating Trials

This experiment was performed to investigate the influence of age on the readiness of *Nv* females to accept *Ng* males as mates and to study whether interspecific mating influences their tendency to re-mate with a conspecific male. We isolated virgin *Nv* females of two age groups: (a) 0–1-d-old, representing newly eclosed females that might have to face mating attempts by *Ng* males inside a multiparasitised host and (b) 2-d-old, representing females that might encounter courting *Ng* males after emergence outside the host. In the first part of the experiment, we observed pairs of one *Nv* female and one *Ng* male for 5 minutes and noted whether they mated or not. Those females that mated with an *Ng* male, were subsequently put together with a conspecific *Nv* male and re-mating events were recorded for another 5 minutes. For control, the same experiment was done using *Nv* males for both matings. The readiness to mate with an *Ng* male and to re-mate with a conspecific was analysed by 2×2 Χ^2^-tests. To investigate whether prior mating with an *Ng* male has any detrimental effects on clutch size and offspring sex ratio we allowed double-mated (first *Ng* and then *Nv*) and single mated *Nv* females (*Nv* only) to parasitise 10 hosts for 48 h. Subsequently, females were removed and hosts were kept at rearing conditions until the next generation emerged. Offspring sex ratios and clutch sizes were determined and compared by a Mann-Whitney *U-*test (n = 20 per treatment).

### Dispersion Experiment

This experiment was performed to investigate whether intra- and interspecific mating influences the dispersion behaviour in *Nv* females. Previous studies have demonstrated that *Nv* females exhibit an increased flight and general locomotor activity after mating [28], [29]. We therefore expected faster dispersion in mated females. As a proxy for natal patch dispersion we recorded the time *Nv* females needed to leave a 150 ml Erlenmeyer flask containing a parasitised host puparium, from which all wasps had emerged as a source of olfactory host cues. Three types of 2-d-old *Nv* females were tested: (a) virgin, (b) mated with an *Nv* male and (c) mated with an *Ng* male (n = 20 per treatment). Females were mated under controlled conditions (treatments b and c) and released singly into open 1.5 ml microcentrifuge tubes together with the empty host puparia. The tubes were transferred into the Erlenmeyer flasks, which were put into transparent plastic boxes (20 cm length×20 cm width×9 cm height). Subsequently, the females were observed for a maximum of 60 min. Dispersion time was defined as the time until the females reached the rim of the Erlenmeyer flask. Dispersion times of virgin and mated females were compared by a Kruskal-Wallis *H-*test followed by Bonferroni-corrected multiple Mann-Whitney *U*-tests.

### Microcosm Experiment

The mate recognition and mating trials described above were performed in small observation chambers and thus interactions between the sexual partners were favoured in these experiments by the experimental design. To study under more realistic conditions whether the presence of *Ng* males on a patch imposes fitness costs on *Nv* females by influencing the offspring sex ratio, we performed a microcosm experiment. For this purpose, we exposed six 2-d-old virgin *Nv* females per replicate for 24 h to two unmated males in a Petri dish. Three combinations of males were chosen, (a) *Nv*+*Nv*, (b) *Nv+Ng* and (c) *Nv*+Ng* (n* = *20 per combination). The asterisk indicates a constrained *Ng* male, which was able to court the females but was prevented from inseminating them by sealing the abdominal tip with a drop of non-toxic super glue. This control experiment was performed to test whether the single unconstrained *Nv* male in treatment (b) is in principle able to sire the same number of offspring than the two *Nv* males in treatment (a) and whether sex ratio effects in treatment (b) are due to the presence of an *Ng* male rather than to sperm depletion of the *Nv* male [30]. After the male-exposure period, females were exposed singly to 10 hosts in a clean Petri dish and allowed to oviposit for 48 h. Offspring sex ratios produced by each female were determined 19–20 days after oviposition. In some replicates, individual females died during the male-exposure or oviposition period. Only those replicates were considered for statistical analysis in which at least three of the females ( = 50% of the six females per replicate) produced offspring. Mean sex ratios were calculated per replicate and treatments were compared by a Kruskal-Wallis *H*-test followed by Holm-corrected multiple Mann-Whitney *U-*tests for individual comparisons. Additionally, we analysed the proportion of all-male broods for the three treatments by Holm-corrected multiple 2×2 Χ^2^ tests.

### Data Accessibility

Original data are available from the Dryad Digital Repository: http://dx.doi.org/10.5061/dryad.n6sk3.

## Results

### Degree of within Host Mating in *Ng* and *Nv*

All *Ng* females collected in the moment of emergence from the host produced female offspring whereas 16 out of 20 *Nv* females produced all-male broods. Hence, the degree of within host mating differed significantly between the studied strains of the two species (Χ^2^ = 26.67, *p*<0.001).

### Pheromone Quantification

**Figure 1:**
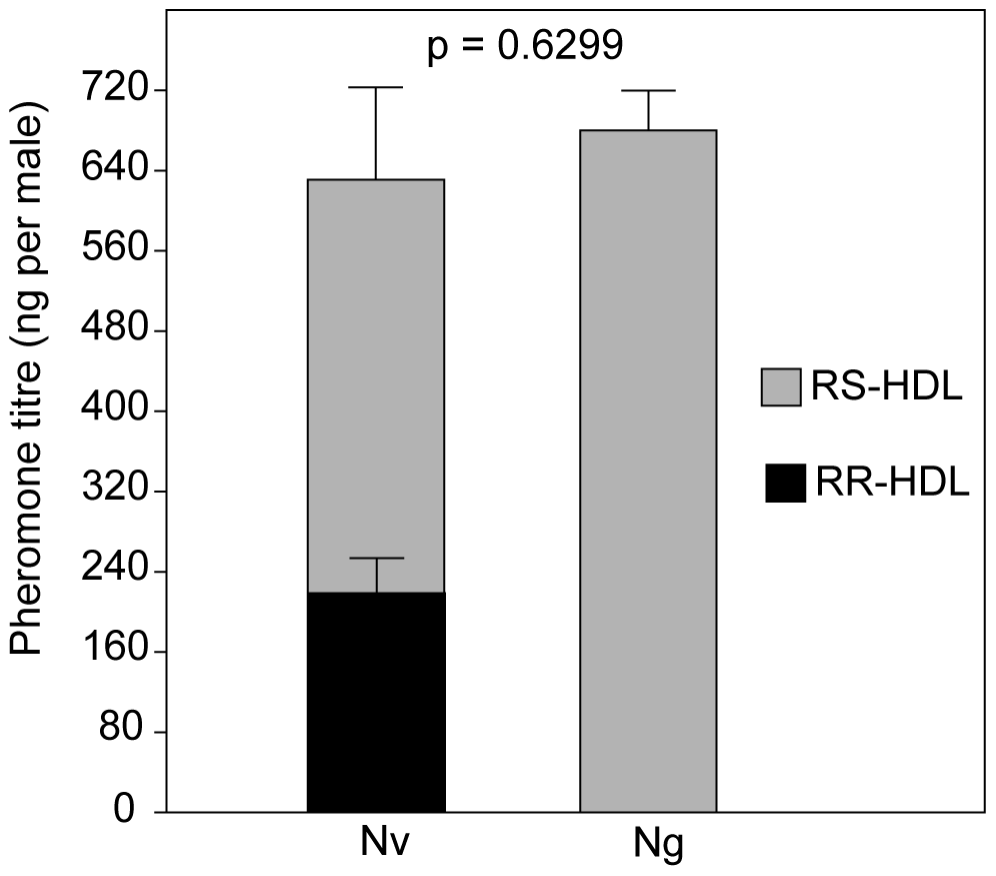
Pheromone titres of male *Nasonia vitripennis* and *N. giraulti*. Mean amounts (+SE) of (4*R*,5*R*)-+(4*R*,5*S*)-HDL 5-hydroxy-4-decanolide (HDL) in abdomen extracts from 2-d-old males of *Nasonia vitripennis* (*Nv*) and *N. giraulti* (*Ng*) (data analysis by a *t*-test for independent samples, n* = *10) (see also Figure S2).

Total HDL amounts found in the extracts of 2-d-old males of both species did not differ significantly (*t* = −0.49021, *p* = 0.6299; Figure 1). Hence, both species invest equally in pheromone production. As previously reported [18], however, the pheromones of *Ng* and *Nv* differ qualitatively with *Ng* producing only *RS* and *Nv* producing both *RS* and *RR*. Like in *Nv* [16], pheromone titres of *Ng* males are age-dependent (Kruskal-Wallis test: *H* = 14.87, *p* = 0.0017, Figure S2). *Ng* males do not have any HDL in their abdomen immediately after eclosion, but pheromone titres increase within the first two days and then remain stable on day three. In 18 out of 20 host puparia from which *Ng* males had emerged, *RS* was undetectable and in two cases it was detected only in very low amounts (19 and 23 ng/host). We therefore conclude that *Ng* males do not use the pheromone immediately after eclosion within the host but rather after emergence outside of the host.

### Pheromone Bioassays

#### Bioassay 1

**Figure 2:**
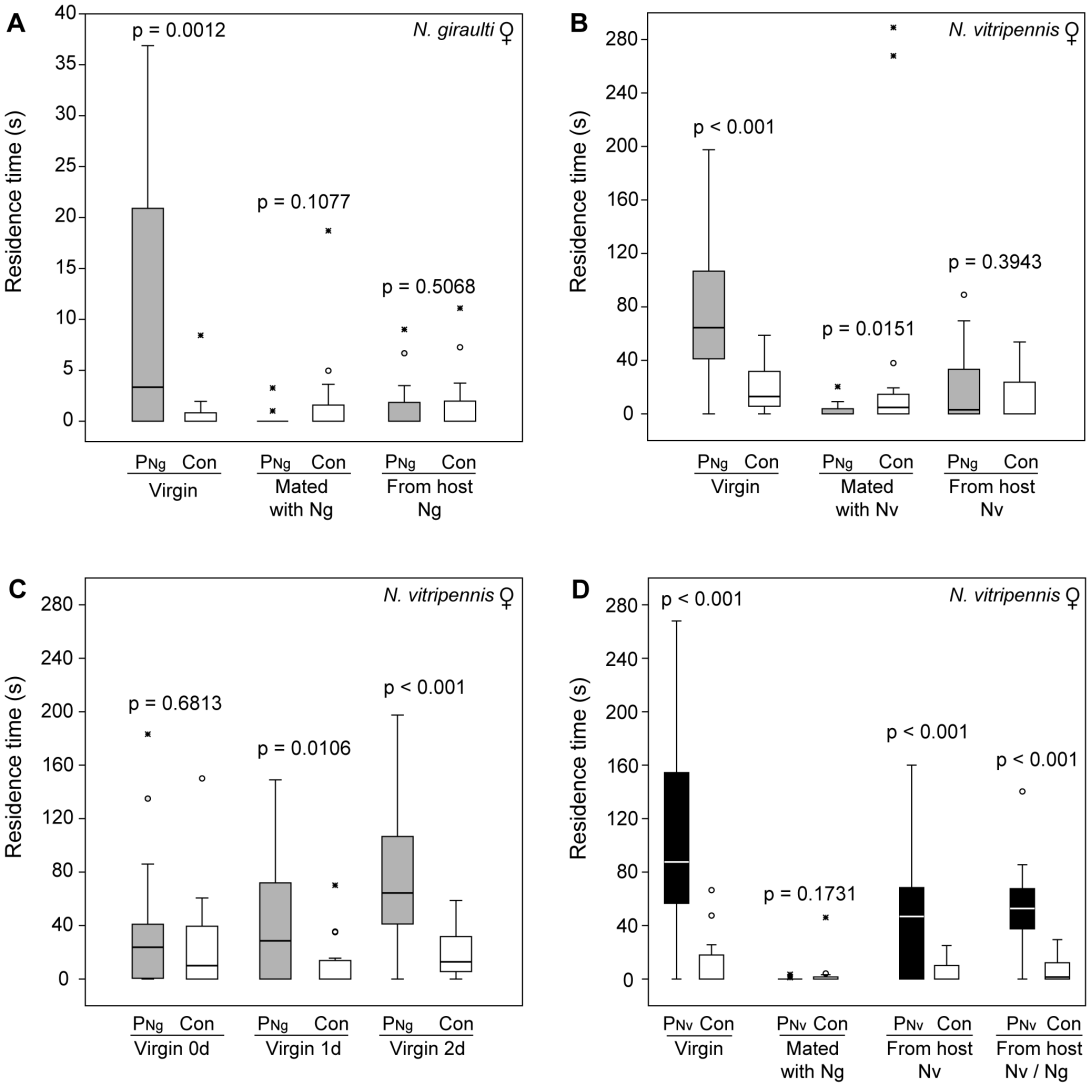
Pheromone response of *Nasonia* females in a two-choice olfactometer. Residence times of (A) *Nasonia giraulti* (*Ng*) and (B–D) *N. vitripennis* (*Nv*) females in the two odour fields of a static two-choice olfactometer (see also Figure S1) when given the choice between a solvent control (Con) and a pheromone extract (representing 100 ng total HDL) of either *Ng* (P*_Ng_*, grey boxes) or *Nv* (P*_Nv_*, black boxes) males. Tested females were either virgin, mated with a con- or heterospecific male under controlled conditions or collected in the moment of emergence from the host. Hosts were either infested by one species only (*Ng*, *Nv*) or multiparasitised by both species (*Ng*/*Nv*). Apart from panel (C), tested females were 1–2 days old. Box-and-whisker plots show median (horizontal line), 25–75 percent quartiles (box), maximum/minimum range (whiskers) and outliers (° >1.5× above box height; * >3× above box height) (data analysis by a Wilcoxon matched-pairs test; n = 20).

*Ng* females were attracted to the conspecific pheromone as virgins (*Z* = 3.237, *p* = 0.0012), whereas mated females (*Z = *1.609, *p* = 0.1077) and those collected in the moment of emergence from the host (*Z = *0.6639, *p* = 0.5068) did not respond (Figure 2*a*). This confirms that the vast majority of *Ng* females mate inside the host and do not need the conspecific pheromone for mate finding outside the host.

#### Bioassay 2

Two-day-old virgin *Nv* females were attracted to the *Ng* pheromone (*Z = *3.421, *p*<0.001), whereas mated *Nv* females (*Z = *2.43, *p* = 0.0151) even avoided the heterospecific pheromone (Figure 2*b*). *Nv* females collected in the moment of emergence from the host were also unresponsive to the heterospecific pheromone (*Z = *0.8519, *p* = 0.3943) although the vast majority of them emerge as virgins (see above). This led us to assume that the response of virgin *Nv* females to the congeneric *Ng* sex pheromone is age-dependent.

#### Bioassay 3

We therefore studied the age dependency of the *Nv* pheromone response under controlled conditions and found that newly eclosed *Nv* females were in fact unresponsive to the *Ng* pheromone (*Z = *0.4107, *p* = 0.6813) but became increasingly responsive after one day (*Z = *2.556, *p* = 0.0106) and two days (*Z = *3.421, *p*<0.001, Figure 2*c*), respectively.

#### Bioassay 4

In contrast to the previous experiment, virgin *Nv* females (*Z = *3.724, *p*<0.001), as well as those collected in the moment of emergence from the host (*Z = *3.845, *p*<0.001), were strongly attracted to the *Nv* pheromone (Figure 2*d*). Hence, *Nv* females discriminate between the heterospecific and the more complex conspecific pheromone blend when leaving the host. Previous experiments have shown that this is enabled by the novel pheromone component *RR* [18]. Remarkably, also *Nv* females which had emerged from multiparasitised hosts containing both *Nv* and *Ng* males were attracted to the conspecific pheromone (*Z = *3.479, *p*<0.001). *Nv* females which had been experimentally mated with *Ng* males outside the host, however, no longer responded to the conspecific pheromone (*Z* = 1.362, *p* = 0.1731) as is the case after mating with conspecifics [16], [19]. This demonstrates that the vast majority of *Nv* females leave their host as virgins even when *Ng* males are present within the host suggesting that they resist possible mating attempts by *Ng* males inside the host. Results of the next experiments demonstrate this explicitly.

### Mate Recognition Tests

**Figure 3:**
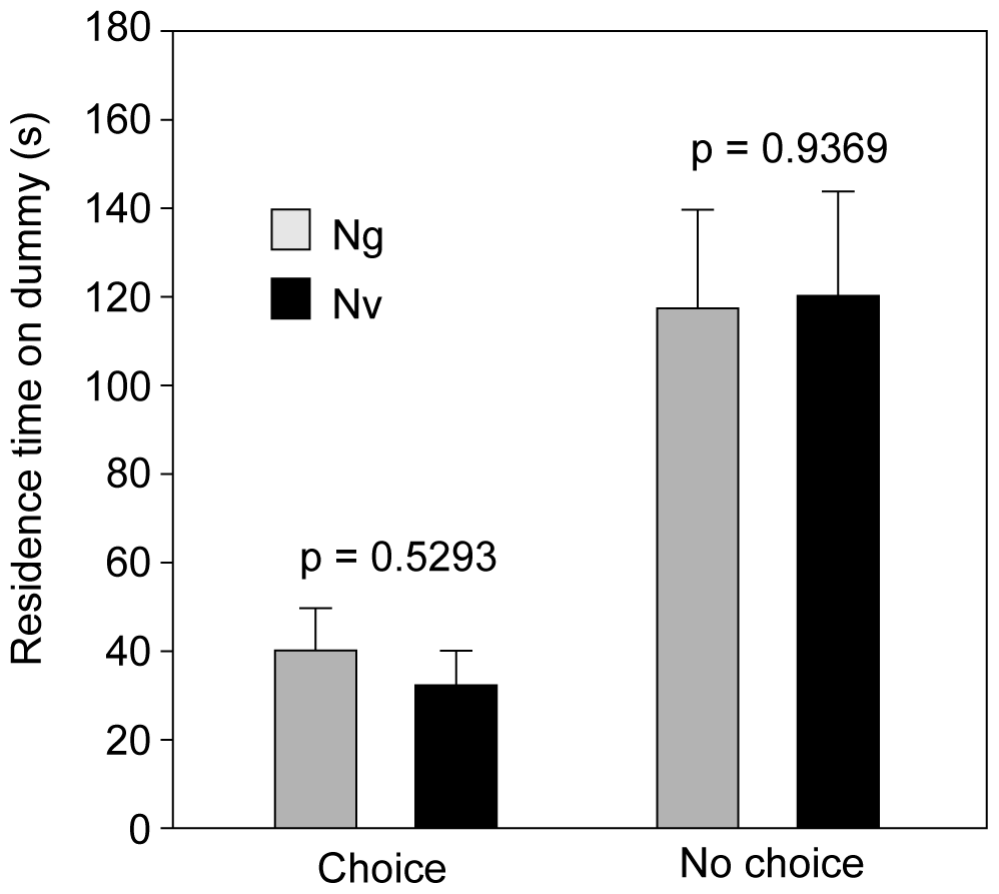
Behavioural response of *Nasonia giraulti* males to female dummies. Mean residence times (+ SE) of *Nasonia giraulti* (*Ng*) males spent on dead *Ng* and *N. vitripennis* (*Nv*) females; female cadavers were offered either simultaneously (choice) or singly (no choice) for five minutes in an observation chamber (data analysis by a *t*-test for independent samples; n* = *20) (see also Figure S3a–b).

*Ng* males neither discriminated between female *Ng* and *Nv* dummies in a choice situation (residence time: *t = *0.6350, *p* = 0.5293; copulation attempts: Χ^2^ = 1.13, *p = *0.4801, Figures 3 and S3 *a*) nor in a no-choice situation (residence time: *t* = −0.0802, *p = *0.9369; copulation attempts: Χ^2^ = 2.67, *p* = 0.1908). Also, the number of dummies mounted first by *Ng* males in the choice experiment was independent of the species (Χ^2^ = 0.10, *p* = 0.7515, Figure S3 *b*). Hence, *Ng* males of our lab strain are equally motivated to engage in courtship with con- and heterospecific females.

### Mating Trials

**Figure 4:**
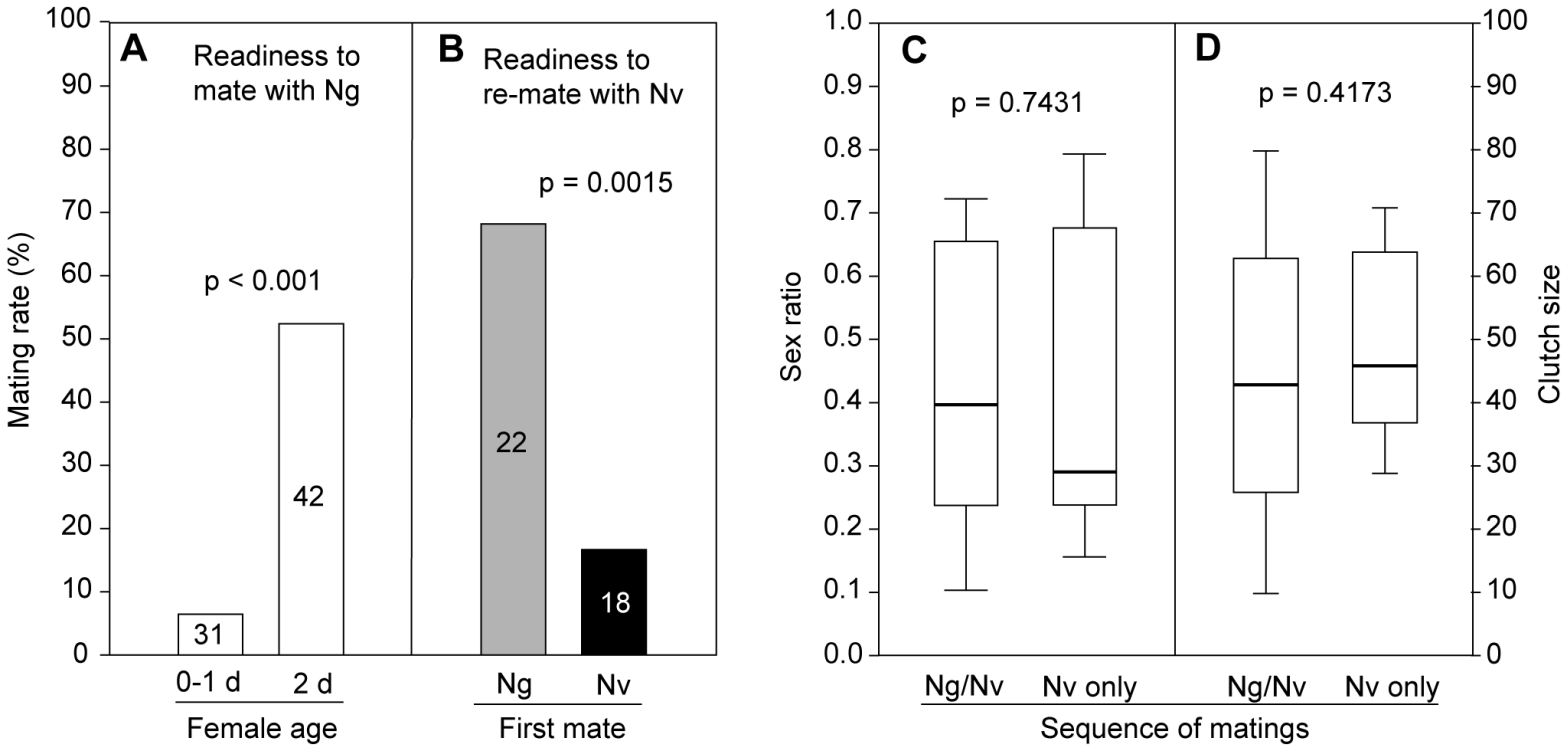
Mating and re-mating behaviour of *Nasonia vitripennis* females and resulting offspring. (A) Proportion of *Nasonia vitripennis* (*Nv*) females of different age that mated with *N. giraulti* (*Ng*) males in the mating trials and (B) proportion of 2-d-old *Nv* females that re-mated with an *Nv* male after a first mating with either an *Ng* or an *Nv* male. Digits on the columns represent numbers of replicates in the individual experiments (data analysis with a 2×2 X^2^-test). (C) Offspring sex ratios and (D) clutch size produced by *Nv* females after sequential double mating with an *Ng* and an *Nv* male (*Ng/Nv*) or after mating with an *Nv* male only (data analysis with a Mann-Whitney *U*-test, n = 20).

Similar to the pheromone responses of *Nv* females (see above), we found an age effect also in their mating behaviour. Only 6.5% of 0–1-d-old *Nv* females were willing to mate with an *Ng* male whereas the proportion of interspecific mating increased to 52% after two days (Χ^2^ = 17.05, *p*<0.001) (Figure 4*a*). Zero to one-day-old *Nv* females are not generally unreceptive because 19 out of 20 signalled receptivity when courted by conspecific males (Χ^2^ = 39.35, *p*<0.001). The tendency of 2-d-old *Nv* females to re-mate with a conspecific male was significantly higher when they had previously mated with an *Ng* male compared to those that had previously mated with an *Nv* male (Χ^2^ = 10.62, *p* = 0.0015; Figure 4*b*). Neither offspring sex ratios (*U* = 133.5, *p* = 0.7431, Figure 4*c*) nor clutch sizes (*U* = 120, *p* = 0.4173, Figure 4*d*) differed between females that had mated twice (first *Ng*, then *Nv*) and those that had mated only once with an *Nv* male. These results indicate that *Nv* females discriminate against *Ng* males much stronger when they are young and the risk of heterospecific mating is high (i.e. after eclosion within the host and shortly after emergence outside the host). Older *Nv* females having erroneously mated with an *Ng* male are principally able to decrease fitness costs by re-mating with a conspecific male.

### Dispersion Experiment

**Figure 5:**
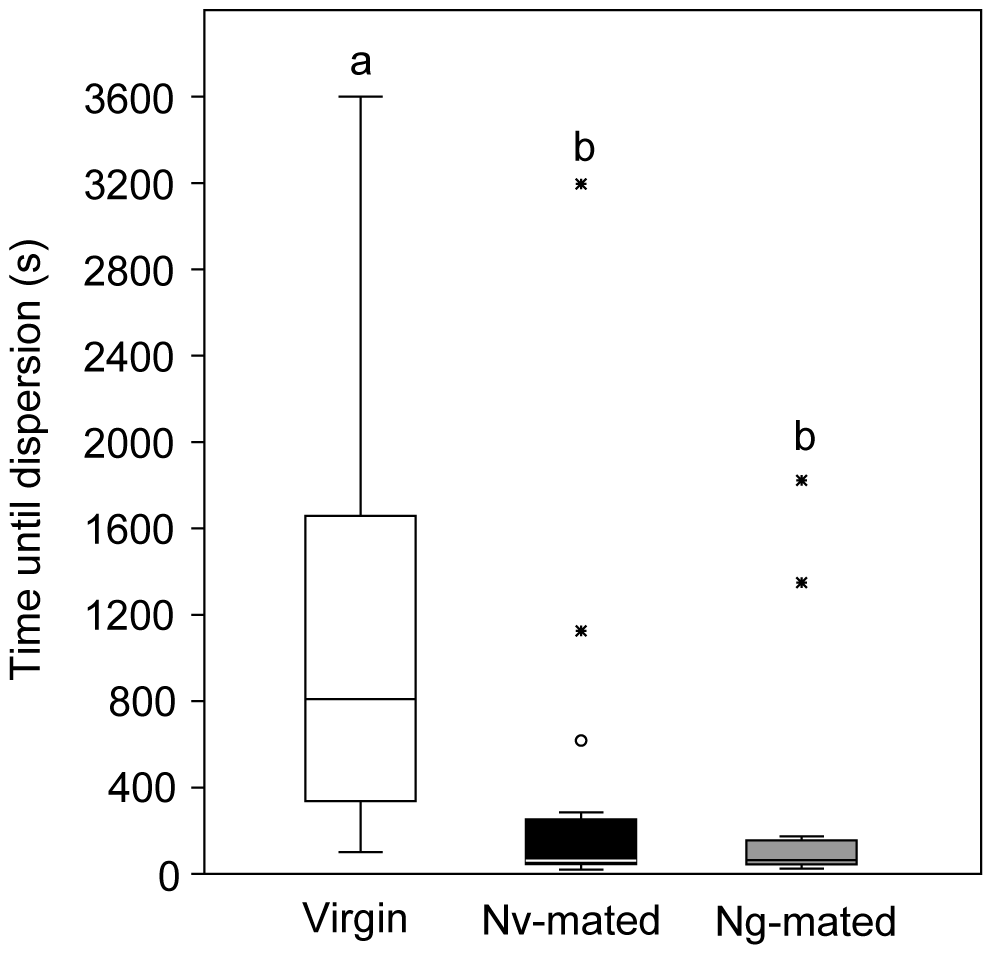
Influence of the mating status on the dispersion behaviour of *Nasonia vitripennis* females. Recorded time needed by virgin and mated (with either an *Nv* or an *Ng* male) *Nv* females to leave an artificial natal host patch. Box-and-whisker plots show median (horizontal line), 25–75 percent quartiles (box), maximum/minimum range (whiskers) and outliers (° >1.5× above box height; * >3× above box height). Different lowercase letters indicate significant differences between treatments at *p*<0.001 (data analysis by a Wilcoxon matched-pairs test; n = 20).

Mated *Nv* females dispersed significantly faster from the artificial natal patch than virgin females irrespective of whether they had mated with a conspecific or heterospecific male (*H = *25.57, *P*<0.001, virgin vs. *Nv-*mated: *U* = 44, *p<*0.001; virgin vs. *Ng-*mated: *U* = 34, *p<*0.001; *Nv* mated vs. *Ng* mated: *U* = 189.5, *p = *0.7867, Figure 5). Consequently, both intra- and interspecific mating equally increase the motivation of *Nv* females to leave the natal patch.

### Microcosm Experiment

**Figure 6:**
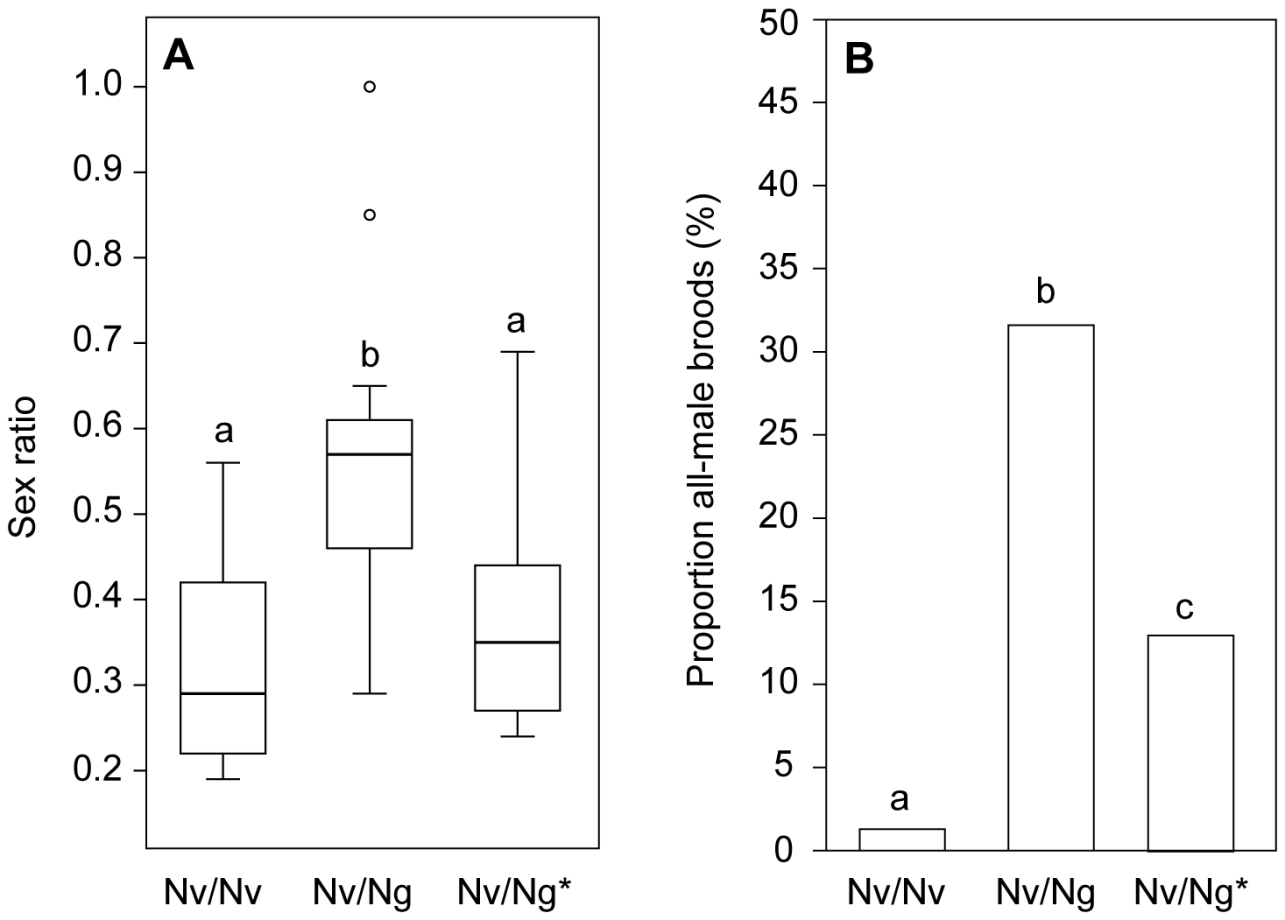
Offspring sex ratios produced by *Nasonia vitripennis* females in the microcosm experiment. (A) Offspring sex ratios and (B) proportion of all-male broods produced by differently treated *Nasonia vitripennis* (*Nv*) females. Prior to oviposition, females were exposed in groups of six individuals to either two *Nv* males (*Nv*/*Nv*), one *Nv* and one *N. giraulti* (*Ng*) male (*Nv*/*Ng*) or one normal and one constrained *Ng* male (*Nv*/Ng*; constrained males were able to perform courtship but were prevented from mating with the females; for this purpose their abdominal tip was sealed with non-toxic glue). Box-and-whisker plots show median (horizontal line), 25–75 percent quartiles (box), maximum/minimum range (whiskers) and outliers (° >1.5× above box height). Different lowercase letters indicate significant differences at p<0.02; data analysis in (A) by a Kruskal-Wallis *H-*test followed by Holm-corrected multiple Mann-Whitney *U*-tests and in (B) by Holm-corrected multiple 2×2 X^2^-tests. (see also Table 1).

The presence of *Ng* males in the microcosm experiment shifted the mean offspring sex ratio produced by *Nv* females in favour of sons (Figure 6*a*, statistical data given in Table 1). This was due to an increased production of all-male broods (Figure 6*b*). A comparison of the *Nv*/*Ng* treatment with the control treatment *Nv*/Ng* allowed differentiation of effects caused by unmated and *Ng-*mated females, respectively. This comparison clearly indicated that a significant number of *Nv* females had mated with the *Ng* male in the *Nv*/*Ng* treatment and that these females had not re-mated with an *Nv* male.

**Table 1:** Statistical differences in the microcosm experiment (Figure 6).

Sex ratio*	Comparison	p-Value
*Nv*/*Nv*	*Nv*/*Ng*	p*<*0.001
*Nv*/*Nv*	*Nv*/*Ng* *	p* = *0.4622
*Nv*/*Ng*	*Nv*/*Ng* *	p* = *0.0033
**All-male broods**		
*Nv*/*Nv*	*Nv*/*Ng*	p<0.001
*Nv*/*Nv*	*Nv*/*Ng* *	p* = *0.0150
*Nv*/*Ng*	*Nv*/*Ng* *	p* = *0.0064

*Kruskal-Wallis test: *H = *20.37, p<0.001.

## Discussion

### Heterospecific Sexual Interactions between *Nv* and *Ng* are Likely to occur in Nature

Based on previous field data [8], [13] and the results presented here, it is reasonable to assume that heterospecific sexual interactions between *Nv* and *Ng* occur in natural habitats. In a comprehensive field study performed in New York State, Grillenberger et al. [13] sampled more than 2000 host pupae from 64 nest boxes at three different regions. In 29% of the sampled nests both *Nv* and *Ng* were found confirming previous data for two other sampling sites [8]. Remarkably, Grillenberger et al. [13] never found *Ng* without *Nv* and in three nests that were analysed in more detail using microsatellites, more than 40% (35 out of 84) of the hosts were multiparasitised by *Nv* and *Ng* [13]. This confirms a previous study stating that *Nv* and *Ng* occur micro-sympatrically over much of their ranges [24] and suggests that *Nv* females and *Ng* males are likely to encounter each other both within the same host habitat and host, respectively. The data presented here demonstrate that *Ng* males of our lab strain do not discriminate between conspecific and *Nv* females during courtship (Figures 3 and S3) suggesting an interspecific interference of CHCs, which are used as mate recognition cues in *Nasonia* and many other parasitic wasps [9], [21], [31]. In a recent study [9], males of another *Ng* lab strain responded even exclusively to heterospecific female dummies (*Nv* and *N. oneida*) and extracts thereof but were unresponsive to conspecifics. Given that mating with *Nv* females results in zero fitness for *Ng* males due to *Wolbachia-*mediated cytoplasmic incompatibility [15], it remained somewhat unclear in the cited study why *Ng* males should evolve to respond to sexual cues from micro-sympatric *Nv* females and ignore conspecific ones. Nonetheless, these results corroborate that *Nv* females are likely to be involved in interspecific sexual interactions with *Ng* males in zones of sympatry.

### Heterospecific Mating is Costly for *Nv* Females

Due to the *Wolbachia*-induced loss of male chromosomes [15], *Ng-*mated *Nv* females produce all-male broods. Hence, females mating with a heterospecific male can principally produce offspring but at clearly suboptimal sex ratios. *Nv* males are flightless and unable to disperse. Therefore, they depend on mating opportunities at the natal patch (local mate competition [12], [32]), which would be missing on patches exploited by a single virgin foundress. Therefore, *Nv* females dispersing after mating with *Ng* only, face the risk of a reproductive dead end. A recent field study performed on two European *Nv* populations demonstrated that host patches exploited by single foundresses are not rare in *Nv*. Five out of 18 nest boxes containing parasitised hosts had been exploited by one female only [10]. Even on host patches parasitised by more than one female, offspring sex ratios are typically female-biased [10], [33] and thus females producing all male broods face fitness costs under this scenario as well.

Our data show that *Nv* females can principally avoid fitness costs due to interspecific mating by re-mating with a conspecific male (Figure 4b). Neither offspring sex ratio nor clutch size differed between re-mated *Nv* females when compared to those mated only once with a conspecific (Figure 4c–d). However, our microcosm experiment suggests that *Ng* males can nonetheless impose fitness costs on *Nv* females because not all of them re-mated in the more spacious environment (Figure 6b). The results of two of our experiments suggest that the degree of intraspecific re-mating might be even lower under natural conditions with females having the chance to disperse after mating. *Ng-*mated *Nv* females (a) no longer respond to the conspecific sex pheromone (Fig. 2d) and (b) show a dramatically increased tendency to disperse from the patch compared to virgin *Nv* females (Fig. 5) corroborating previous studies that demonstrated a mating-induced increase of flight and general locomotor activity in *Nv* females [28], [29]. Hence, despite the general motivation of mismated *Nv* females to re-mate with a conspecific male, their re-mating rate in nature might be much lower than in our lab experiments, in which encounters with conspecific males were enforced by the experimental design. Therefore, field tests are necessary to determine the re-mating rate of *Ng-*mated *Nv* females under natural conditions.

### Why do *Ng* Males Invest in the Production of a Sex Attractant?

Our results demonstrate that *Ng* males produce as much HDL as *Nv* males even though the stereochemical composition differs between both species (Fig. 1). Hence, *Nv* and *Ng* invest equal amounts of linoleic acid (LA) in the production of the sex attractant. The twofold unsaturated fatty acid LA is the precursor of HDL biosynthesis in *Nasonia* [27]. Polyunsaturated fatty acids including LA affect the sperm production in many animals [34] and the availability of LA has been shown to co-vary with fertility in *Nv* [27]. This suggests a trade-off between pheromone and sperm production in *Nasonia*. Therefore, the high investment of *Ng* males in pheromone production is somewhat surprising given that they inseminate most of their females within the host where a sex attractant is dispensable. Furthermore, mated *Ng* females and those collected in the moment of emergence from the host did not respond to the conspecific pheromone in our experiments (Fig. 2a). There are two possible explanations for these findings. First, *Ng* males might invest in pheromone biosynthesis to attract and mate with females of the competing sympatric species *Nv* to increase the reproductive success of conspecific females inseminated previously by them within the host. However, individual fitness benefits for *Ng* males pursuing this strategy are difficult to conclude because *Ng* and *Nv* females inseminated by a given *Ng* male would need to arrive at the same host patch after dispersal and compete there for the same hosts. This is somewhat unlikely and furthermore, *Ng-*mated *Nv* females, albeit constrained to produce all-male broods, are able to produce offspring, which, like dual sex broods, might compete with the *Ng* offspring for nutritional resources after multiparasitism. An alternative explanation for the investment of *Ng* males in pheromone production might be within host mating ratios below 100% as found previously in field populations of *Ng* [8]. Thus *Ng* males, after having inseminated the majority of females within the host, might nevertheless invest in the production of pheromones to attract the few remaining conspecific females that emerge as virgins outside the host.

### Age-dependent Behavioural Plasticity Reduces the Risk of Interspecific Mating Costs

Young virgin *Nv* females are particularly prone to become involved in interspecific sexual interactions. They might encounter courting *Ng* males inside multiparasitised hosts and outside in the moment when leaving the host. Our data demonstrate that young *Nv* females have evolved counter-adaptations for both situations. First, they are significantly less likely than older females to signal receptiveness towards courting *Ng* males (Figure 4a). Secondly, young *Nv* females do not respond to the *Ng* sex attractant when leaving the host (Figure 2b) although most of them emerge as virgins. Both of these behaviours are real discrimination rather than a general lack of responsiveness in young *Nv* females because they accept conspecific males as mates (see results of the mating trials) and respond to the more complex *Nv* sex pheromone when leaving the host (Figure 2d). In a recent study, Niehuis et al. [18] have demonstrated that it is the newly evolved pheromone component *RR*, which is responsible for the ability of *Nv* females to discriminate between pheromone markings of conspecific and heterospecific males. The addition of synthetic *RR* to the two-component pheromone blend of *Ng* males rendered the blend more attractive for *Nv* females whereas *Ng* females did not discriminate between the two pheromone phenotypes [18].

The question as to why *Nv* females become less choosy with increasing age is puzzling because in contrast to other insects showing an age-dependent decrease of choosiness (see for example [35]–[38]) they cannot gain any benefits from mating with *Ng* at any time of their life. We suggest that, in a natural environment, the probability is low that females would reach an age two days without getting inseminated because mating occurs in *Nasonia* typically shortly after emergence [39]. Therefore, the decreased choosiness of older females found in our lab experiments might not result in significant fitness consequences in nature.

### Proposed Scenario of Pheromone Evolution in *Nasonia*

**Figure 7:**
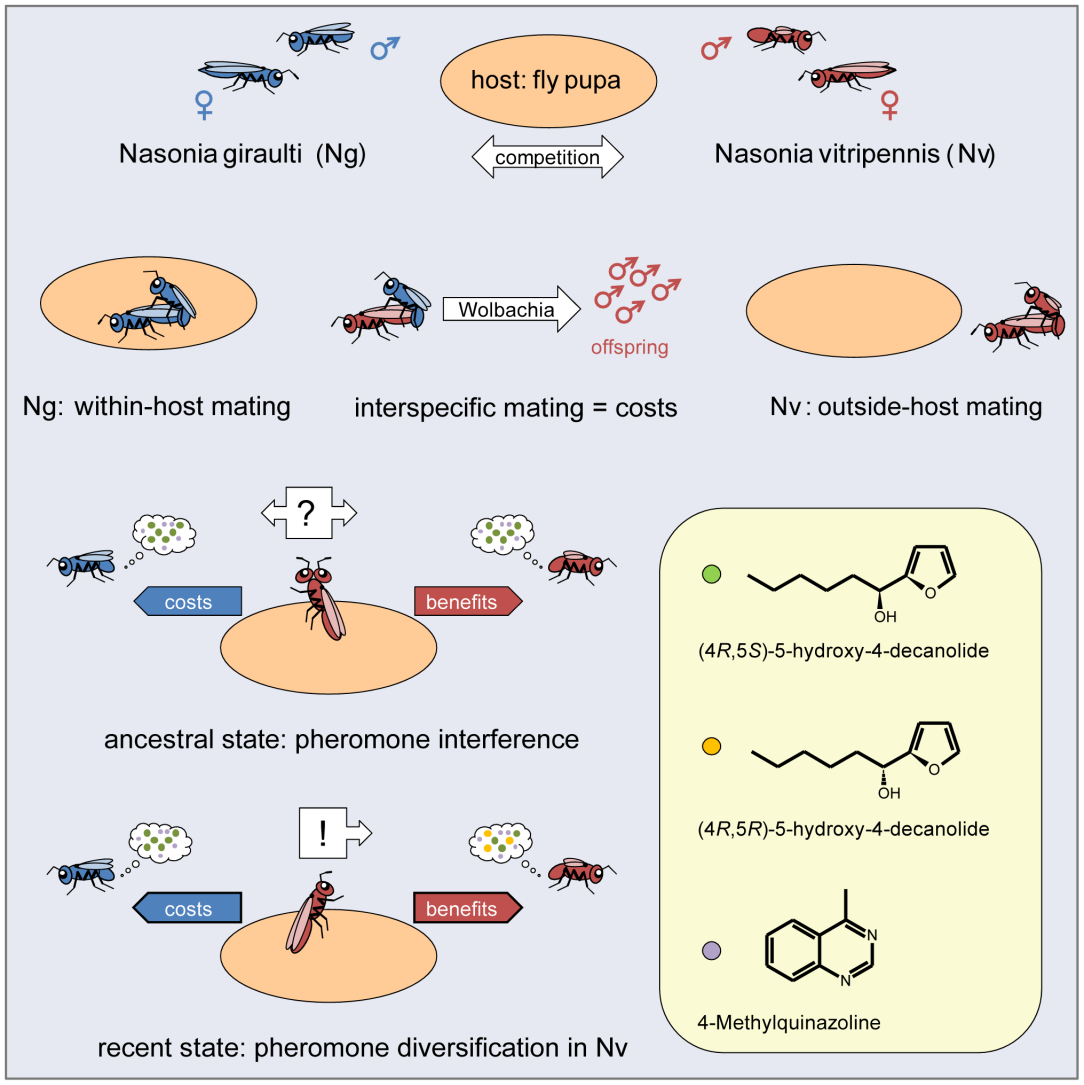
Hypothetical scenario of pheromone evolution in *Nasonia*. For details see text.

Based on the results presented here and in a previous study [18], we propose the following scenario of pheromone divergence in *Nasonia* (Figure 7). Pheromone use and mating outside the host are likely features of the ancestral mating strategy in *Nasonia*, because males of all known *Nasonia* species and the closely related species *Trichomalopsis sarcophagae* produce two-component sex pheromones (*RS*+MQ) [16]. The switch of *Ng* to a very high ratio of within host mating made the sex pheromone widely dispensable in this species. We exclude that *Ng* males use the pheromone inside the host because newly emerged males do not possess it and it was undetectable in the vast majority of hosts from which males had emerged. However, *Ng* males did not abolish the chemical signal during evolution but continued to invest in pheromone production to the same degree as *Nv* presumably to attract the low proportion of conspecific females emerging as virgins. The resulting signal interference between *Nv* and *Ng* in ancestral times and the fitness costs resulting from heterospecific mating might have been selective forces driving the evolution of the reported age-dependent mate discrimination in *Nv* and the diversification of the male sex attractant (*RR* as a novel pheromone component). As a final step, *Nv* females have evolved the chemosensory adaptations to exploit the novel three component sex pheromone (for explanations of the suggested order of signal diversification and chemosensory adaptation see [18]).

From the data currently available, it is impossible to infer whether pheromone diversification in *Nv* has occurred before or after the *Wolbachia*-mediated reproductive isolation of the *Nasonia* species. In the former case it might have been part of the speciation process and thus represents a case of reinforcement, i.e., the evolution of prezygotic isolation by natural selection against the production of unfit hybrids [40] (according to Butlin [40], the term reinforcement should be restricted to cases where hybridisation is still possible). The involvement of reinforcement in speciation is difficult to demonstrate, but its signature can be detected by showing that reproductive barriers between two taxa are stronger in zones of contact than in allopatry [41]. As for the male sex pheromone in *Nasonia,* all allopatric European and sympatric North American *Nv* populations studied so far produce the more complex three-component pheromone blend (RR/RS/MQ) [18]. However, as Servedio & Noor emphasise [3], the signature of reinforcement may be easily erased by the spread of premating isolation mechanisms into areas of allopatry. Hence, the more complex pheromone blend of *Nv* might have evolved in zones of contact with *Ng* before *Nv* has spread around the world. This would support the hypothesis that the cosmopolitan species *Nv* originates in North America rather than in the Old World, a question that is still under debate [42]. It has to be emphasised, however, that the outlined scenario, albeit supported by a comprehensive set of behavioural data, is a hypothetical one. Alternative evolutionary processes such as genetic drift or sexual selection cannot be fully excluded and might have led just as well to the pheromone diversification in *Nv.*

## Conclusions

The study of pheromone evolution is probably one of the most challenging tasks chemoecologists are facing today [2], [31], [43]. The present study has implications for our understanding of this process by providing evidence for the hypothesis that communication interference of sympatric species using similar sexual signals can generate selective pressures that lead to their divergence [2]. However, our data are hitherto based on two inbred lab strains only and the motivation for interspecific mating has been shown to vary significantly between geographic *Nasonia* populations [24]. Hence, future studies using outbred lab strains [44] or field populations are needed to confirm the findings of the present study before substantial conclusions on the origin of pre-zygotic isolation in *Nasonia* wasps can be drawn.

## Supporting Information

Figure S1 **Schematic view of the two-choice olfactometer used for the pheromone bioassays.**
(TIF)

Figure S2 **Age dependency of the pheromone titre of individual** ***Nasonia giraulti*** **males.** Box-and-whisker plots show median (horizontal line), 25–75 percent quartiles (box), maximum/minimum range (whiskers). Different lowercase letters indicate significant differences between age groups at p<0.05 (data analysis by Kruskal-Wallis *H-*test and Mann-Whitney *U*-tests, n = 5 per age group).
(TIF)

Figure S3 **Behavioural response of** ***Nasonia giraulti*** **males to** **female dummies.** (A) Proportion of *Nasonia giraulti* (*Ng*) males showing copulation attempts with the dead *Ng* and *N. vitripennis* (*Nv*) females offered either simultaneously (choice) or singly (no choice) for five minutes in an observation chamber (data analysis by Fisher’s exact test; n = 20). (B) Proportion of first mounts of *Ng* males in the choice experiment (data analysis by a Χ^2^ test for the goodness of fit).
(TIF)

## References

[1] Groning J, Hochkirch A (2008) Reproductive interference between animal species. Quart Rev Biol 83: 257–282 10.1086/590510 18792662

[2] Smadja C, Butlin RK (2009) On the scent of speciation: The chemosensory system and its role in premating isolation. Heredity 102: 77–97 10.1038/hdy.2008.55 18685572

[3] Servedio MR, Noor MAF (2003) The role of reinforcement in speciation: Theory and data. Annu Rev Ecol Evol System 34: 339–364 10.1146/annurev.ecolsys.34.011802.132412

[4] Wyatt, TD. (2003) Pheromones and Animal Behaviour. Communication by Smell and Taste. Cambridge: Cambridge University Press, 391 p.

[5] El-Sayed AM (2013) The Pherobase: Database of Pheromones and Semiochemicals. Available: http://www.pherobase.com/. Accessed: 23 January 2014.

[6] Raychoudhury R, Desjardins CA, Buellesbach J, Loehlin DW, Grillenberger BK, et al (2010) Behavioral and genetic characteristics of a new species of *Nasonia* . Heredity 104: 278–288 10.1038/hdy.2009.147 20087394 PMC3533498

[7] Werren JH, Richards S, Desjardins CA, Niehuis O, Gadau J, et al (2010) Functional and evolutionary insights from the genomes of three parasitoid *Nasonia* species. Science 327: 343–348 10.1126/science.1178028 20075255 PMC2849982

[8] Darling DC, Werren JH (1990) Biosystematics of *Nasonia* (Hymenoptera, Pteromalidae) - 2 new species reared from birds nests in North-America. Ann Entomol Soc Am 83: 352–370.

[9] Buellesbach J, Gadau J, Beukeboom LW, Echinger F, Raychoudhury R, et al (2013) Cuticular hydrocarbon divergence in the jewel wasp *Nasonia*: Evolutionary shifts in chemical communication channels? J Evol Biol 26: 2467–2478 10.1111/jeb.12242 24118588 PMC3809909

[10] Grillenberger BK, Koevoets T, Burton-Chellew MN, Sykes EM, Shuker DM, et al (2008) Genetic structure of natural *Nasonia vitripennis* populations: Validating assumptions of sex-ratio theory. Mol Ecol 17: 2854–2864 10.1111/j.1365-294X.2008.03800.x 18482258

[11] Hamilton WD (1967) Extraordinary sex ratios. Science 156: 477–488 10.1126/science.156.3774.477 6021675

[12] Werren JH (1983) Sex-ratio evolution under local mate competition in a parasitic wasp. Evolution 37: 116–124 10.2307/2408180 28568021

[13] Grillenberger BK, van de Zande L, Bijlsma R, Gadau J, Beukeboom LW (2009) Reproductive strategies under multiparasitism in natural populations of the parasitoid wasp *Nasonia* (Hymenoptera). J Evol Biol 22: 460–470 10.1111/j.1420-9101.2008.01677.x 19210592

[14] Shuker DM, West SA (2004) Information constraints and the precision of adaptation: Sex ratio manipulation in wasps. Proc Natl Acad Sci USA 101: 10363–10367 10.1073/pnas.030804101 15240888 PMC478577

[15] Breeuwer JAJ, Werren JH (1990) Microorganisms associated with chromosome destruction and reproductive isolation between two insect species. Nature 346: 558–560 10.1038/346558a0 2377229

[16] Ruther J, Stahl LM, Steiner S, Garble LA, Tolasch T (2007) A male sex pheromone in a parasitic wasp and control of the behavioral response by the female's mating status. J Exp Biol 210: 2163–2169 10.1242/jeb.02789 17562890

[17] Ruther J, Steiner S, Garbe LA (2008) 4-methylquinazoline is a minor component of the male sex pheromone in *Nasonia vitripennis* . J Chem Ecol 34: 99–102 10.1007/s10886-007-9411-1 18085340

[18] Niehuis O, Büllesbach J, Gibson JD, Pothmann D, Hanner C, et al (2013) Behavioural and genetic analyses of *Nasonia* shed light on the evolution of sex pheromones. Nature 494: 345–348 10.1038/nature11838 23407492

[19] Steiner S, Ruther J (2009) How important is sex for females of a haplodiploid species under local mate competition? Behav Ecol 20: 570–574 10.1093/beheco/arp033

[20] Tanner ME (2002) Understanding nature's strategies for enzyme-catalyzed racemization and epimerization. Acc Chem Res 35: 237–246 10.1021/ar000056y 11955052

[21] Steiner S, Hermann N, Ruther J (2006) Characterization of a female-produced courtship pheromone in the parasitoid *Nasonia vitripennis* . J Chem Ecol 32: 1687–1702 10.1007/s10886-006-9102-3 16900425

[22] van den Assem J, Jachmann F, Simbolotti P (1980) Courtship behavior of *Nasonia vitripennis* (Hym., Pteromalidae): some qualitative, experimental evidence for the role of pheromones. Behaviour 75: 301–307 10.1163/156853980X00456

[23] Ruther J, Thal K, Blaul B, Steiner S (2010) Behavioural switch in the sex pheromone response of *Nasonia vitripennis* females is linked to receptivity signalling. Animal Behaviour 80: 1035–1040 10.1016/j.anbehav.2010.09.008

[24] Bordenstein SR, Drapeau MD, Werren JH (2000) Intraspecific variation in sexual isolation in the jewel wasp *Nasonia* . Evolution 54: 567–573 10.1111/j.0014-3820.2000.tb00059.x 10937233

[25] Bordenstein SR, Werren JH (1998) Effects of A and B Wolbachia and host genotype on interspecies cytoplasmic incompatibility in *Nasonia* . Genetics 148: 1833–1844. 9560398 10.1093/genetics/148.4.1833 PMC1460083

[26] Drapeau MD, Werren JH (1999) Differences in mating behaviour and sex ratio between three sibling species of *Nasonia* . Evol Ecol Res 1: 223–234.

[27] Blaul B, Ruther J (2011) How parasitoid females produce sexy sons: a causal link between oviposition preference, dietary lipids and mate choice in *Nasonia* . Proc R Soc Lond B 278: 3286–3293 10.1098/rspb.2011.0001 PMC3169019 21429922

[28] King B (1993) Flight activity in the parasitoid wasp *Nasonia vitripennis* (Hymenoptera: Pteromalidae). J Ins Behav 6: 313–321 10.1007/BF01048112

[29] King BH, Grimm KM, Reno HE (2000) Effects of mating on female locomotor activity in the parasitoid wasp *Nasonia vitripennis* (Hymenoptera : Pteromalidae). Environ Entomol 29: 927–933 10.1603/0046-225X-29.5.927

[30] Ruther J, Matschke M, Garbe LA, Steiner S (2009) Quantity matters: Male sex pheromone signals mate quality in the parasitic wasp *Nasonia vitripennis* . Proc R Soc Lond B 276: 3303–3310 10.1098/rspb.2009.0738 PMC2817171 19535374

[31] Ruther J (2013) Novel insights into pheromone-mediated communication in parasitic hymenopterans. In: Wajnberg E, Colazza S, editors. Chemical Ecology of Insect Parasitoids. Chichester: Wiley. 112–144.

[32] Werren JH (1980) Sex ratio adaptations to local mate competition in a parasitic wasp. Science 208: 1157–1159 10.1126/science.208.4448.1157 17783073

[33] Burton-Chellew MN, Koevoets T, Grillenberger BK, Sykes EM, Underwood SL, et al (2008) Facultative sex ratio adjustment in natural populations of wasps: Cues of local mate competition and the precision of adaptation. Am Nat 172: 393–404 10.1086/589895 18710342

[34] Wathes DC, Abayasekara DRE, Aitken RJ (2007) Polyunsaturated fatty acids in male and female reproduction. Biol Reprod 77: 190–201 10.1095/biolreprod.107.060558 17442851

[35] Moore PJ, Moore AJ (2001) Reproductive aging and mating: the ticking of the biological clock in female cockroaches. Proc Natl Acad Sci USA 98: 9171–9176 10.1073/pnas.161154598 11470903 PMC55392

[36] Tinghitella RM, Weigel EG, Head M, Boughman JW (2013) Flexible mate choice when mates are rare and time is short. Ecol Evol 3: 2820–2831 10.1002/ece3.666 24101975 PMC3790532

[37] Mautz BS, Sakaluk SK (2008) The effects of age and previous mating experience on pre- and post-copulatory mate choice in female house crickets (*Acheta domesticus* L.). J Ins Behav 21: 203–212 10.1007/s10905-008-9120-9

[38] Klein AL, Trillo MC, Albo MJ (2012) Sexual receptivity varies according to female age in a Neotropical nuptial gift-giving spider. J Arachnol 40: 138–140.

[39] van den Assem J, Gijswijt MJ, Nübel BK (1980) Observations on courtship strategies and mating strategies in a few species of parasitic wasps (Chalcidoidea). Neth J Zool 30: 208–227.

[40] Butlin R (1987) Speciation by reinforcement. Trends Ecol Evol 2: 8–13 10.1016/0169-5347(87)90193-5 21227808

[41] Marshall JL, Arnold ML, Howard DJ (2002) Reinforcement: The road not taken. Trends Ecol Evol 17: 558–563 10.1016/S0169-5347(02)02636-8

[42] Raychoudhury R, Grillenberger BK, Gadau J, Bijlsma R, van de Zande L, et al (2010) Phylogeography of *Nasonia vitripennis* (Hymenoptera) indicates a mitochondrial-*Wolbachia* sweep in North America. Heredity 104: 318–326 10.1038/hdy.2009.160 20087396

[43] Steiger S, Schmitt T, Schaefer HM (2010) The origin and dynamic evolution of chemical information transfer. Proc R Soc Lond B 278: 970–979 10.1098/rspb.2010.2285 PMC3049038 21177681

[44] van der Zande L, Ferber S, de Haan A, Beukeboom L, van Heerwaarden J, et al. (2013) Development of a *Nasonia vitripennis* outbred laboratory population for genetic analysis. Mol Ecol Resour 11: (in press). doi: 10.1111/1755-0998.12201. 10.1111/1755-0998.12201 PMC4260118 24215457

