# Supplementary figures and images for: Pheromone Diversification and Age-Dependent Behavioural Plasticity Decrease Interspecific Mating Costs in Nasonia
Source: PLoS One. 2014 Feb 14;9(2):e89214. doi: 10.1371/journal.pone.0089214 (PMC3925242; doi:10.1371/journal.pone.0089214)

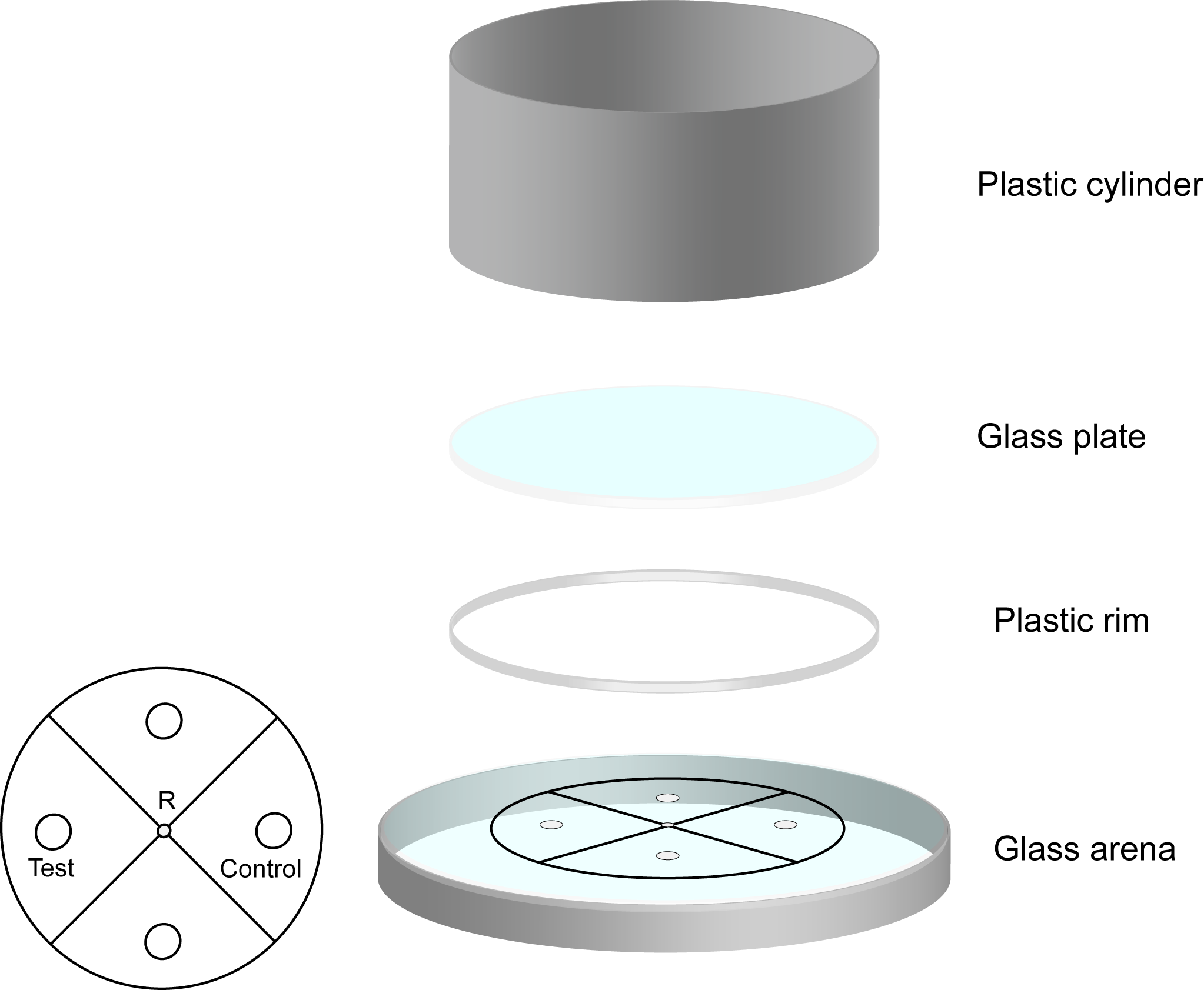

Supplement: Figure S1 — Schematic view of the two-choice olfactometer used for the pheromone bioassays. (TIF) [file pone.0089214.s001.tif]

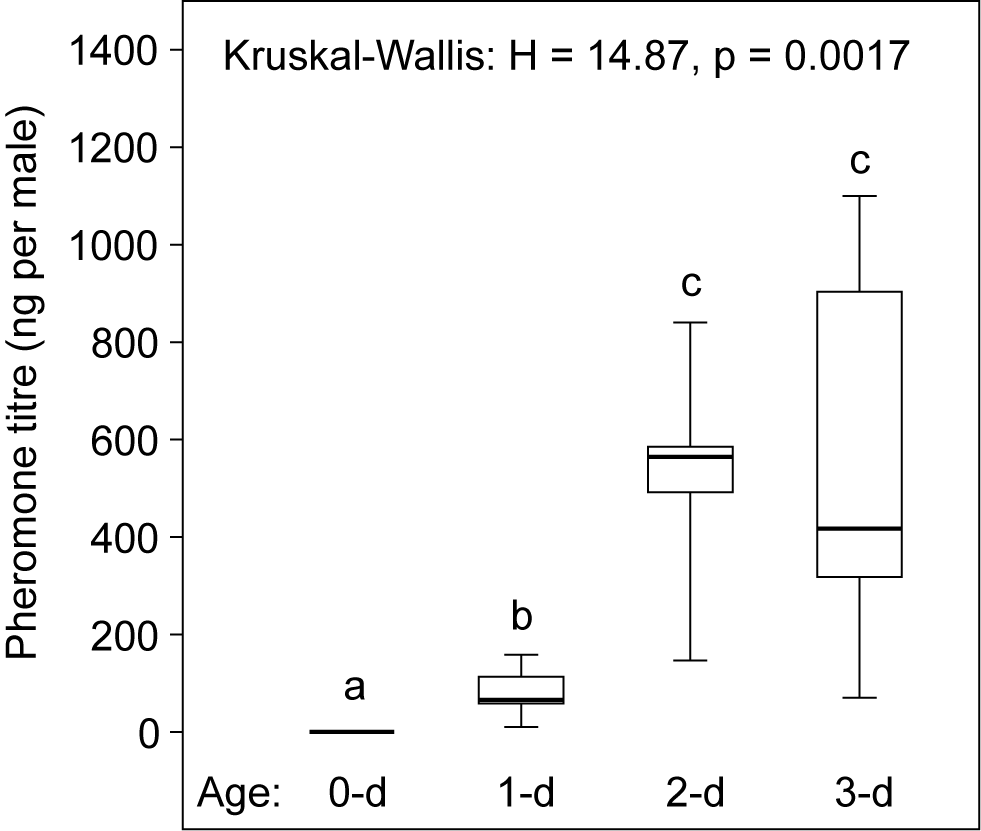

Supplement: Figure S2 — Age dependency of the pheromone titre of individual Nasonia giraulti males. Box-and-whisker plots show median (horizontal line), 25–75 percent quartiles (box), maximum/minimum range (whiskers). Different lowercase letters indicate significant differences between age groups at p<0.05 (data analysis by Kruskal-Wallis H-test and Mann-Whitney U-tests, n = 5 per age group). (TIF) [file pone.0089214.s002.tif]

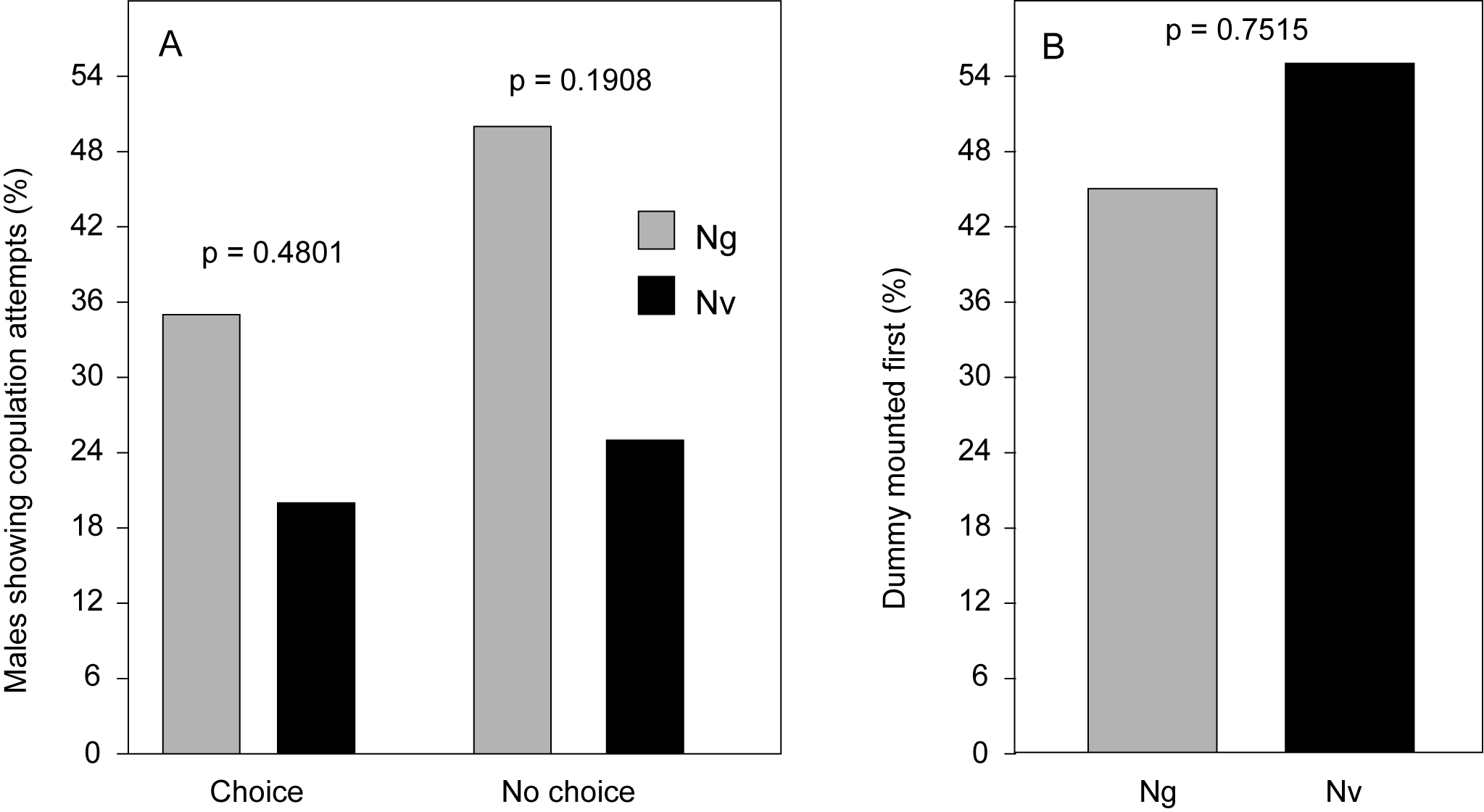

Supplement: Figure S3 — Behavioural response of Nasonia giraulti males to female dummies. (A) Proportion of Nasonia giraulti (Ng) males showing copulation attempts with the dead Ng and N. vitripennis (Nv) females offered either simultaneously (choice) or singly (no choice) for five minutes in an observation chamber (data analysis by Fisher’s exact test; n = 20). (B) Proportion of first mounts of Ng males in the choice experiment (data analysis by a Χ2 test for the goodness of fit). (TIF) [file pone.0089214.s003.tif]
